# Left sided diverticulitis presenting as a right lumbar fistula: a case report

**DOI:** 10.4076/1757-1626-2-7146

**Published:** 2009-08-04

**Authors:** Barnabas Rigden Green, Vickram Joypaul

**Affiliations:** Department of General Surgery, South Tyneside NHS Foundation TrustHarton Lane, South Shields, South Tyneside, NE34 0PLUK

## Abstract

The formation of fistulae is a recognised complication of diverticulitis. This case report describes sigmoid diverticulitis presenting as a right psoas abscess with a colocutaneous fistula. The report highlights the role of appropriate imaging and a high index of suspicion in anyone presenting with a discharging lumbar abscess, especially when the focus of infection may be from a contra-lateral source.

## Introduction

Acquired (propulsion type) diverticulae are herniations of colonic mucosa through the muscular wall of the bowel at points where blood vessels penetrate the colonic wall [1]. Diverticulosis is rare in the African and Asian populations however is found in approximately 25% of the Western population over the age of 40 who undergo a barium enema [2] and its incidence is thought to be on the increase [3]. Diverticulosis in itself is usually inconsequential however it may become problematic; presenting as uncomplicated diverticulitis (often managed non-operatively) or complicated diverticulitis (acute diverticulitis accompanied by abscess, obstruction, free intra-abdominal perforation or fistulation). Fistulation occurs in approximately 5% of cases and can arise spontaneously and post-operatively. Sites of fistulation are colovesical, colovaginal, enterocolic and colocutaneous and rarer sites of colocutaneous fistulation have been described [4,5]. This report presents an unusual site for fistulation in a case of complicated left sided diverticulitis.

## Case presentation

**Figure 1.**
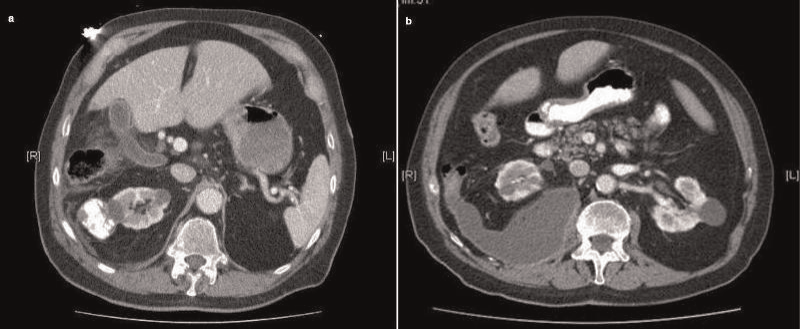
Calcified right renal cyst **(a)**, right psoas abscess **(b)**.

An 81-year-old British Caucasian male presented to the physicians with a cough, haemoptysis and an irregularly irregular pulse rhythm. A CT chest and upper abdomen demonstrated consolidation of the right lung base and he was treated for his pneumonia and atrial fibrillation. Shortly after admission he developed abdominal pain, diarrhoea and a low-grade pyrexia. His CT scan was reviewed and it was noted that there was a small amount of intra-abdominal fluid with several retroperitoneal lymph nodes. The right kidney had an atypical area of calcification within its upper pole and he was subsequently referred to the urologists for further evaluation (Figure 1a). As his condition failed to improve he underwent a further CT scan that demonstrated a large right psoas abscess (Figure 1b). This was drained under CT guidance and he was eventually discharged with a urology outpatient follow up as it was thought his renal problem was the cause of the abscess. Upon review the patient was symptomatically better, a diagnosis of an old calcified cyst in the right kidney was made and he was discharged to his GP.

**Figure 2.**
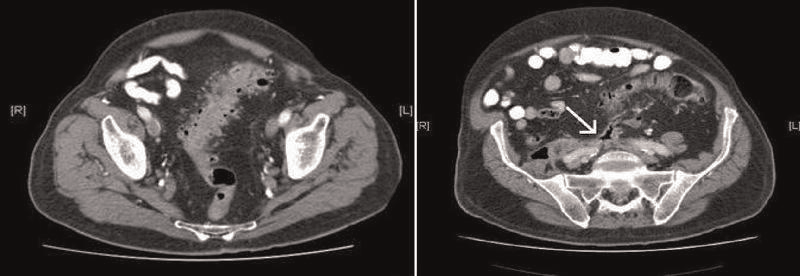
Sigmoid diverticulosis **(a)**, fistulous tract (arrow) to abscess **(b)**.

Eight months later, after a permanent pacemaker had been fitted, an interval USS showed no residual psoas abscess. However, one month subsequently he was admitted under the general surgeons with a 3-week history of mild back pain, diarrhoea and rigors. He also had a discharging abscess in his right lumbar region which contained gas bubbles. A CT scan revealed sigmoid diverticulosis (Figure 2a) with a recurrence of the extensive right-sided retroperitoneal abscess and a tract connecting it to the sigmoid colon (Figure 2b).

As an interim measure whilst being optimised for surgery, the abscess was drained with a 14F pigtail drain. At laparotomy the sigmoid colon was found frozen in the pelvis with perforated sigmoid diverticulosis noted to be the cause of the right sided retroperitoneal abscess. A sigmoid colectomy and primary anastomosis was performed and he was treated post-operatively with antibiotics. He made a slow but uneventful recovery and was discharged fit enough to undergo an open cholecystectomy for acute cholecystitis 16 months later.

## Discussion

Diverticulosis of the colon covers a wide clinical spectrum. Most patients will remain asymptomatic throughout their lifetime and will require no particular intervention or follow-up [6]. In the remaining population who develop symptoms, one quarter will develop diverticular haemorrhage and the remaining three quarters will develop diverticulitis [7]. Approximately one quarter of patients with diverticulitis will develop potentially life-threatening complications including perforation, fistulae, obstruction or a stricture [8]. Disease specific classifications have been developed to attempt to predict outcome and in diverticulitis; the Hinchey classification (Table 1) is the most widely used [9,10]. Several studies have documented the mortality rates for patients with diverticulitis and abscess formation (Hinchey I/II) at 5-10% and patients with perforated disease with peritonitis (Hinchey III/IV) at 20-50% [11,12,13].

**Table 1.** Hinchey and Modified Hinchey classifications of diverticulosis

Hinchey classification
Stage I Pericolic abscess or phlegmon
Stage II Pelvic, intra-abdominal or retroperitoneal abscess*
Stage III Generalised purulent peritonitis
Stage IV Faecal peritonitis
*Modified Hinchey classification
Stage IIa Distant abscess amenable to surgical drainage
Stage IIb Complex abscess associated with/without fistula

With respect to management the current literature agrees with historical literature in that patients with uncomplicated diverticulitis may be managed as an outpatient with dietary modification and oral antibiotics providing they can be followed up and have no appreciable fever, vomiting or marked peritonitis [14]. If these conditions are not met, or the patient fails to improve, they should be hospitalised and managed with intravenous antibiotics and dietary modification [15]. (Evidence III; Grade of Recommendation: B). In complicated disease with abscesses >2 cm, radiologically guided percutaneous drainage is usually the most appropriate course of treatment [10]. (Evidence III; Grade of Recommendation: B). Peritonitis forms an indication for emergency surgery and elective surgery is reserved for fistula closure and obstruction [16]. The two main procedures in the emergency setting are a Hartmann’s procedure (HP) and primary resection and anastomosis (PRA). A recent systematic review has demonstrated overall mortality rates of 15.1% and 4.9% respectively [17]. and would therefore seem to favour PRA in the emergency setting. However, recent evidence from a multi-institutional study of patients with perforated diverticulitis causing generalized peritonitis has reported mortality rates of 3% when managed with laparoscopic peritoneal lavage [18]. The recommendation of elective resection in a patient who has had attacks of uncomplicated diverticulitis needs a careful risk-benefit assessment as recent evidence has indicated that prophylactic resection would have little impact in preventing subsequent complications [19].

This case of acute diverticulitis presents with an unusual site of fistulation in a patient with previously undiagnosed diverticulosis. It highlights the significant morbidity which can ensue from the complications of diverticulitis and the important role of CT scanning in these patients. It also demonstrates the difficulty in diagnosis in some presentations and serves as a reminder to have a high index of suspicion in the aetiology of a psoas abscess, especially one on the contra-lateral side to the disease.

The first difficulty in diagnosis surrounds the patients’ co-morbidity. In hindsight it appears the patient had his first attack of acute diverticulitis whilst hospitalised for pneumonia. It is possible that the antibiotics used to treat the chest masked a localised diverticular perforation which later presented with the first psoas abscess. The second difficulty in diagnosis surrounds the right renal lesion which was initially thought to be malignant and possibly a cause of the psoas abscess. However, the bacteria grown from the abscess was *Citrobacter youngae*, a bacterium found in human stool. With the suspicion of a colonic origin, perhaps this case should have been referred to the colorectal surgeons for further assessment. The third area of difficulty in diagnosis is the site of the abscess (right) not being thought to be connected to the contra-lateral bowel. It was the presence of gas bubbles in the abscess on the second admission that alerted the surgeons to a potentially colonic origin and this was confirmed by CT scanning.

## Conclusion

This unusual presentation of a colocutaneous fistula as a complication of acute diverticulitis highlights the need to have a high index of suspicion when presented with a psoas abscess, particularly one discharging onto the surface with visible gas bubbles in it, the need to examine all the surrounding evidence carefully and the role of CT scanning in acute complicated diverticulitis. It is also a testimony to careful preoperative management and successful surgery in a patient with significant co-morbidity.

## Abbreviations

CT: Computer tomography
F: French
GP: General practitioner
HP: Hartmann’s procedure
PRA: Primary resection and anastomosis
USS: Ultrasound scan

## References

[bib-001] Morson BC (1975). Pathology of diverticular disease of the colon. Clin Gastroenterol.

[bib-002] Mortensen NJMcC, Jones O, Russell RCG, Williams NS, Bulstrode CJK (2004). From The small and large intestines. Bailey & Love’s Short Practice of Surgery.

[bib-003] Subramanian S, Tinto A, Majeed A, Melville DM, Hart AR, Morris CR, Maxwell JD, Kang JK (2002). Colonic diverticulitis: a disease on the rise. Gut.

[bib-004] Fazio VW, Church JM, Jagelman DB, Weakley GL, Lavery IC, Rarazi R, vanHillo M (1987). Colocutaneous fistulas complicating diverticulitis. Dis Colon Rectum.

[bib-005] Drabble EH, Greatorex RA (1994). Colocutaneous fistula between the sigmoid colon and popliteal fossa in diverticulosis. Br J Surg.

[bib-006] Stollman N, Raskin JB (2004). Diverticulosis of the colon. Lancet.

[bib-007] Young-Fadok RM, Roberts PL, Spencer MP, Wolff BG (2000). Colonic diverticulosis. Curr Probl Surg.

[bib-008] Kang JY, Melville D, Maxwell JD (2004). Epidemiology and management of diverticulosis of the colon. Drugs Aging.

[bib-009] Hinchey EJ, Schaal PG, Richards GK (1978). Treatment of perforated diverticulosis of the colon. Adv Surg.

[bib-010] Sher M, Agachan F, Bortal M, Nogueras JJ, Weiss EG, Wexner SD (1997). Laparoscopic surgery for diverticulitis. Surg Endosc.

[bib-011] Berry AR, Turner WH, Mortensen NJMcC, Kettlewell MGW (1989). Emergency surgery for complicated diverticulosis. A five-year experience. Dis Colon Rectum.

[bib-012] Tudor RG, Farmakis N, Keighley MRB (1994). National audit of complicated diverticulosis: analysis of index cases. Br J Surg.

[bib-013] Elliot TB, Yego S, Irvin TT (1997). Five-year audit of the acute complications of diverticulosis. Br J Surg.

[bib-014] Ambrosetti P (1997). Diverticulitis of the left colon. Recent advances in Surgery.

[bib-015] Rafferty J, Shellito P, Hyman NH, Buie WD (2006). Standards Committee of American Society of Colon and Rectal Surgeons. Practice parameters for sigmoid diverticulitis. Dis Colon Rectum.

[bib-016] Szojda MM, Cuesta MA, Mulder CM, Felt-Bersma RJ (2007). Management of Diverticulitis. Aliment Pharmacol Ther.

[bib-017] Constantinidas VA, Tekkis PP, Athanasiou T, Aziz O, Purkayastha S, Remzi FH, Fazio VW, Aydin N, Darzi A, Senapti A (2006). Primary resection with anastomosis *vs.* Hartmann’s procedure for nonelective surgery for acute colonic diverticulitis: a systematic review. Dis Colon Rectum.

[bib-018] Myers E, Hurley M, O’Sullivan GC, Kavanagh D, Wilson I, Winter DC (2008). Laparoscopic peritoneal lavage for generalized peritonitis due to perforated diverticulitis. Br J Surg.

[bib-019] Janes SEJ, Meagher A, Frizelle FA (2005). Elective surgery after acute diverticulitis. Br J Surg.

